# Validation of Fecal Glucocorticoid Metabolites as Non-Invasive Markers for Monitoring Stress in Common Buzzards (*Buteo buteo*)

**DOI:** 10.3390/ani14081234

**Published:** 2024-04-19

**Authors:** Lara-Luisa Grundei, Tanja E. Wolf, Florian Brandes, Karolin Schütte, Fritjof Freise, Ursula Siebert, Chadi Touma, Michael Pees

**Affiliations:** 1 Department of Small Mammal, Reptile and Avian Medicine and Surgery, University of Veterinary Medicine Hannover, Foundation, Bünteweg 9, 30559 Hanover, Germany; 2 Department of Behavioral Biology, School of Biology/Chemistry, Osnabrück University, Barbarastrasse 11, 49076 Osnabrück, Germany; 3 Mammal Research Institute, Faculty of Natural and Agricultural Sciences, University of Pretoria, Pretoria 0028, South Africa; 4 Wildlife Rescue and Conservation Center, Hohe Warte 1, 31553 Sachsenhagen, Germany; 5 Department of Biometry, Epidemiology and Information Processing, University of Veterinary Medicine Hannover, Foundation, Bünteweg 2, 30559 Hanover, Germany; 6 Institute of Terrestrial and Aquatic Wildlife Research, University of Veterinary Medicine Hannover, Foundation, Bischofsholer Damm 15, 30173 Hanover, Germany

**Keywords:** fGCM, hormone, behavior, rehabilitation, animal welfare, bird of prey, wildlife, corticosterone

## Abstract

**Simple Summary:**

The measurement of stress in wildlife, especially using non-invasive methods, is an important tool when monitoring wild animals taken into human care. However, the methods used to measure stress hormone metabolites from feces must be validated for each species. Eight Common Buzzards habituated to humans were placed into prepared aviaries and their feces were collected over a period of seven days. For biological validation, handling and restraint were used as a stress event. Hormone metabolites were analyzed using three different enzyme immunoassays to find the most suitable one. In addition, a degradation experiment was conducted to find out how long the fecal glucocorticoid metabolites (fGCMs) remained stable in the feces at room temperature. The cortisone enzyme immunoassay detected a distinctive peak of excreted glucocorticoid metabolites in response to the stress event. We found no significant differences between the sexes, but a diurnal variation in the stress hormone metabolites. Immunoreactive metabolite concentrations showed a significant change eight hours after defecation, indicating degradation processes. Our study successfully validated the non-invasive measurement of fGCMs as a stress indicator in the Common Buzzard and could lay the foundation for future studies providing new insights for animal welfare research in this species.

**Abstract:**

For wild animals, being in captivity in wildlife centers can cause considerable stress. Therefore, it is necessary to establish and validate non-invasive tools to measure chronic stress during rehabilitation. Eight Common Buzzards which lived in permanent husbandry were placed individually into prepared aviaries and their feces were collected before, during and after a stress event for biological validation over a period of seven days. The extracted fecal glucocorticoid metabolites (fGCMs) were analyzed with three different enzyme immune assays (EIA) to find the most suitable one. Additionally, we aimed to investigate the stability of fGCM levels after defecation because further metabolization by bacterial enzymes can lead to changed results. The Cortisone-EIA performed best in males and females and showed that the stress event led to an fGCM increase of 629% (557% in females and 702% in males) in relation to basal values. We found no significant differences between the sexes, but observed significant differences between different times of day. FGCM concentration significantly changed after eight hours at room temperature. Our study successfully validated the non-invasive measurement of fGCM as a stress indicator in Common Buzzards and could therefore lay the foundation for future studies providing new insights for animal welfare research in Buzzards.

## 1. Introduction

Wildlife centers are institutions for the treatment and rehabilitation of injured, diseased, or orphaned wildlife and aim to release healthy individuals back into nature [1]. The human custody of wild animals nevertheless causes considerable stress for the individual animal [2,3,4]. Chronic stress is linked to immunodeficiency and increased susceptibility to diseases [5,6,7,8,9]. Therefore, the individual animal’s welfare should always be the highest priority [1].

Birds of prey only show insufficient objectively measurable external signs of stress, such as immobility or inappetence [3]. In Germany, all species of raptors are protected by law, which means that any stalking, capturing or killing of an individual is strictly prohibited [10]. Exceptions are made for temporary keeping for health care by qualified persons and permanent keeping with an exceptional permit for hunting, research or breeding purposes [10]. Furthermore, birds of prey are important as the top link of the food chain as so-called “sentinel species”, as most pathogens and environmental contaminants in the prey animals are accumulated and reflected in them [11]. Their role in research has thus become more and more important because they are massively influenced by human intervention in nature, e.g., expansion of agricultural land use, urbanization, and illegal hunting [12].

To date, there is a lack of scientific data on human-induced stress in birds of prey in wildlife centers [3]. Therefore, investigating stress during rehabilitation and establishing tools to measure chronic stress are important for developing guidelines to improve animal welfare in wildlife centers [13].

Stress responses in the organism are significantly influenced by the hypothalamic–pituitary–adrenal axis (HPA axis) [7,14,15]. In this endocrinological control circuit, the hypothalamus in the brain produces corticotropin-releasing hormone (CRH), which is passed on to the anterior pituitary gland (adenohypophysis) [7,14]. There, adrenocorticotropic hormone (ACTH) is produced under the influence of CRH and is transported via the bloodstream to the adrenal cortex, where it stimulates the synthesis of corticosteroids like cortisol and corticosterone [7,14]. In birds, in contrast to most mammals, corticosterone plays a far greater role than cortisol [7]. Corticosteroids are hormones from the group of glucocorticoids and they supply glucose to the body through catabolic metabolic processes, influence fat and protein metabolism, the formation of blood cells, and the immune system [7,15]. The body reacts to stress with an increase in cortisol and corticosterone levels, which react with a time delay and last longer compared to catecholamines such as noradrenaline [16].

Steroid hormones can be measured by different methods, such as ptilochronology [17] or blood sampling [18]. Especially during the growth phase, corticosterone deposits can be extracted from the feathers and linked to fitness and pigmentation, as described for *Buteo buteo* [19] and other birds of prey [17,20,21,22]. Plasma corticosterone levels are the most reliable parameter for visualizing past stress and have been successfully measured in other *Buteo* species [23]. For a long time, this invasive technique was common practice, but it is only suitable to a limited extent for determining stress hormone concentrations in wild animals, since capture, handling, and the sampling itself can have an influence on hormone concentrations [24]. For this reason, as well as from an animal welfare perspective, it is recommended to use non-invasive methods [25].

Since steroid hormones are ultimately metabolized in the liver and excreted in urine and feces, the corresponding metabolites, including glucocorticoid metabolites, can be determined in the animals’ excreta [16]. Measuring fecal glucocorticoid metabolites (fGCMs) has become increasingly important in recent years and was already successfully carried out in numerous mammalian species [18,26,27]. Methods for measuring fGCMs have also been established for some bird species in the past, e.g., Great Tit [28], European Starling [29], European Stonechat [30], Domestic Chicken [31,32], Humboldt Penguin [33], and Budgerigar [34]. A physiological stress response can be determined in fresh fecal samples collected without any animal contact. However, since each species may have a different excretion and metabolite pattern of fGCMs, careful validation must be carried out for each of the species studied, which proves the applicability of the method and provides comparative values for the assessment of the values measured in the subsequent application [18,26]. The study design for the collection of fecal samples and processing also strongly depends on the animal species and must be developed individually. Validation can be performed either “physiologically” by administering pharmacological agents such as ACTH or dexamethasone, or “biologically” by measuring stress hormone metabolites during particularly challenging situations [26].

Since physiological validation is not always possible for endangered species, and since this also means additional stress for the animal due to capture, handling, and injection, biological validation is the method of choice to show the activity of the adrenocortical activity [26]. By collecting fecal samples before, during, and after a stressful event, a curve of the fGCMs can be derived, from which a basal value can be determined [26]. At least one of the two methods has been applied to some American raptor species like the Northern Spotted Owl [35], California Spotted Owl [36,37], Barred Owl, Great Horned Owl [38], Golden Eagle, Peregrine Falcon [39], American Kestrel [40], Red-Tailed Hawk [4], and Red-Shouldered Hawk [41]. In Europe, only the Northern Goshawk [42] and the White-tailed Sea Eagle [43] have been studied for stress hormone metabolites in urine or feces, but none of the most common representatives of raptors found in Europe have been studied in this manner [44,45].

The aim of this study was to validate the method of non-invasive stress measurement in the most common bird of prey in Germany, the Common Buzzard (*Buteo buteo*), in order to establish a protocol for determining fGCM concentrations in animals temporarily kept in wildlife centers.

Additionally, we aimed to investigate the stability over time of fGCM levels after defecation at room temperature. Owing to the fact that bacterial enzymes can further metabolize the stress hormones in the non-frozen feces, this can lead to higher or lower cross-reactive metabolites and, thus, to altered results [26,46].

## 2. Materials and Methods

### 2.1. Pre-Study

To select a suitable assay and test the sampling method, a pre-study with one female and one male Common Buzzard was conducted. The birds had lived in permanent husbandry and were therefore expected to already have become acclimatized to captivity and human proximity. After they were brought to the Wildlife Rescue and Conservation Center (Sachsenhagen, Germany), they were placed individually in quietly situated aviaries, which were approx. 3 × 4 m in size and 2.50 m high, closed on three sides with a barred door. One to two crossbars at different heights served as perches. The floor was lined with pond liner and attached to the back wall halfway up by means of a plastic pipe and a clamping bar to catch the droppings that were emitted from a high perch. The animals were fed daily at 12:00 with day-old chicks, chicken breast/wings, or rats. They had a large, shallow bowl of water available at all times.

After a habituation period of two weeks, fecal samples were collected in fecal collection tubes (CVet Kotprobenröhrchen, Covetrus DE GmbH, Düsseldorf, Germany) for 7 days; from day 2 to 6, sampling took place every two hours from 08:00 to 24:00.

All dropped feces without uric acid were collected and immediately frozen at −18 °C until analyses.

The “stress event” on day 4 at 12:00 was standardized as a capture, veterinary examination with X-ray and blood sampling, and restraint in a transport box. Each bird was caught with a landing net and briefly handled. The animals were then placed in a transport box (a plastic dog kennel with a barred door) and carried to the main building of the wildlife center for examination. The birds were weighed, their body condition score (BCS) was estimated by palpation of the sternum, and external examinations were carried out to check for injuries and ectoparasites. In the case that pressure marks were found on the feet, these were classified according to the grading for pododermatitis, i.e., grades I to V [47]. Grade I describes an erythema and flattened papillae, grade II indicates hyperkeratosis and inflammation, grade III is used for deep necrosis and hyperkeratosis, grade IV describes the involvement of soft tissue leading to tendosynovitis, and grade V is used to indicate the involvement of skeletal structures like osteomyelitis. In addition, an X-ray and a blood test were performed to determine the birds’ health status. At 14:00 the birds were returned to the aviaries. In total, the duration of the handling procedure from capture until release lasted about 120 min for each bird.

The frozen samples were extracted after drying in a hot air oven (U40, Memmert GmbH & Co. KG, Schwabach, Germany) for 48 h at 80 °C. For this purpose, the samples were mortared and 0.05 g of the fecal powder was mixed with 3 mL of 60% methanol [48,49,50]. If the sample amount was less than 0.05 g, correspondingly less methanol was added. Samples with less than 0.02 g dried weight were excluded from the study [51]. After 30 min of shaking on a vibrating plate (Köttermann GmbH, Hänigsen, Germany) and 20 min of centrifugation at 4500 rpm (Z306, HERMLE Labortechnik GmbH, Wehingen, Germany), the supernatants were transferred to a 2 mL tube (Eppendorf Safe-Lock Tubes, Eppendorf SE, Hamburg, Germany) and refrozen. The extracts were sent to the Laboratory of the Department of Behavioral Biology at the University of Osnabrück, Germany, on dry ice for analysis.

To identify a suitable enzyme immunoassay (EIA), the samples were analyzed for immunoreactive fGCM concentrations using three different assays: (i) 11-oxoaetiocholanolone II EIA (detecting fGCMs with a 5β-3α-ol-11-one structure), (ii) Cortisone EIA (measuring 4-pregnene-17 α, 21-diol-3,11,20-trione-21-HS), and (iii) 5α-pregnane-3β, 11β,21-triol-20-one EIA (detecting 3β,11β-diol-CM). Detailed assay characteristics, including cross-reactivities, are described by Möstl et al. (2002) for the 11-oxoaetiocholanolone II EIA, by Rettenbacher et al. (2004) for the Cortisone assay, and by Touma et al. (2003) for the 5α-pregnane-3β, 11β,21-triol-20-one EIA [31,52,53]. All assays were in-house assays, as described elsewhere, and were performed on microtiter plates following established protocols [54].

### 2.2. Main Study

To confirm that the chosen assay indicated stress from human intervention and to establish basal values, six Common Buzzards (three females, three males) from permanent husbandry were chosen for the main study. Owing to the fact that there were only three identical aviaries available, the sampling of the six Buzzards took place in two rounds lasting four weeks each.

Three weeks before sampling started, the animals were placed in the aviaries described above to acclimatize them to their new surroundings. After one week, the wildlife center staff started entering the aviary several times a day without taking samples so that the birds became used to this procedure. In week 4, a sampling interval of four hours was introduced, sampling taking place between 08:00 and 24:00 from day 1 to day 7. The temperature in the aviaries corresponded to the outside temperature, which was consistently cool and sometimes damp (approx. 5 to 16 °C) in the months of February and March.

The collection method, the stress event, and the extraction and analysis protocols were analogous to the pre-study.

The animal experiment was approved by the Lower Saxony State Office for Consumer Protection and Food Safety (LAVES, trial application no. TV 21A577).

### 2.3. Stability of Fecal Glucocorticoid Metabolite Concentrations Post-Defecation

To investigate the stability of fecal glucocorticoid metabolite concentration after defecation, we collected fresh fecal samples (*n* = 42) of various adult Common Buzzards, which had been brought to wildlife centers (Wildlife Rescue and Conservation Center, Sachsenhagen, Germany; Wildtierhilfe Deutschland, Burgwedel, Germany) and the clinic (Department of Small Mammal, Reptile and Avian Medicine and Surgery, University of Veterinary Medicine Hannover, Foundation, Hanover, Germany) due to injuries or weakness. Samples were collected immediately after defecation (up to 60 min later) and frozen until further processing. After all samples were thawed at room temperature, they were mixed thoroughly by hand in a beaker with a wooden spatula and divided into four sets with eight subsamples each, which were placed on a foiled surface at room temperature (approx. 20 °C). The samples were re-collected and frozen after 0 h (control), 1 h, 2 h, 4 h, 8 h, 12 h, 24 h, and 48 h, respectively, and stored at −18 °C until analysis.

### 2.4. Statistical Analysis

Data evaluation was performed with the software Microsoft Excel 2016 (Microsoft Corporation, Redmond, WA, USA) and SAS Enterprise Guide 7.1 and SAS software 9.4 (SAS Institute Inc., Cary, NC, USA).

The data were log-transformed to obtain a normal distribution. Differences in sexes, days, and time of day were examined with a linear mixed model with main effects as mentioned above and day-time-of-day interaction to model the times on specific days. Individual variation was modeled using a random effect for the animals and the correlation over time due to repeated measurements by an autoregressive correlation structure. For post hoc pairwise comparisons, Tukey–Kramer adjustment was used. The baseline values of females and males were averaged (mean value) from metabolite concentrations from all samples except those from 0 to 16 h after the stress event. The peak of the stress event was displayed in percentage in relation to the baseline (set at 100%). Differences in alteration between the control sample of the stability experiment and the samples collected at 1 to 48 h post-defecation were examined with Analysis of Variance (ANOVA) allowing for unequal variances in groups and the Dunnett’s test for post hoc comparison. A significance was assumed with *p* ≤ 0.05.

## 3. Results

### 3.1. Assay Validation

Of the three tested enzyme immunoassays (EIAs) the Cortisone-EIA was the most responsive for both the female and the male. The other two EIAs also detected an increase in fGCM concentration after the stress event, but the stress response was most evident in the Cortisone assay. Intra-assay coefficient of variations, determined by repeated measurements of high- and low-value quality controls, were 5.7% and 4.7% for the 11-oxoaetiocholanolone II EIA, 7.5% and 9.0% for the Cortisone EIA, and 5.1% and 5.5% for the 5α-pregnane-3β, 11β,21-triol-20-one EIA, respectively. The inter-assay coefficient of variation, only determined for the Cortisone EIA by repeated measurements of high- and low-value quality controls, was 9.8% and 9.6%, respectively. After identifying the most suitable EIA with regard to fGCM elevation after the stress event, the entire subsequent sample set was assessed using only the Cortisone EIA.

### 3.2. Stress Response

**Figure 1:**
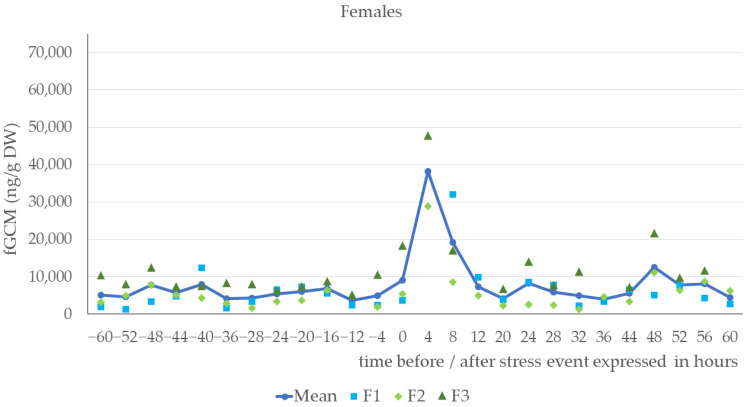
Fecal glucocorticoid metabolite (fGCM) concentrations in ng/g dry weight (DW) of female Buzzards: F1, F2, F3 (*n* = 3) before and after stress event. Missing data points are due to missing or insufficient fecal samples.

**Figure 2:**
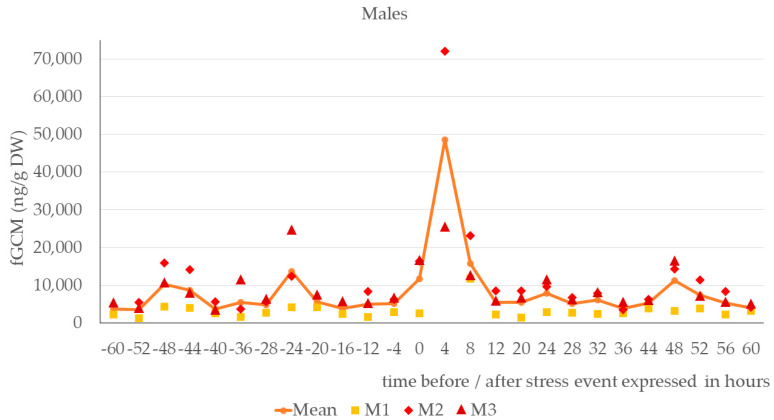
Fecal glucocorticoid metabolite (fGCM) concentrations in ng/g dry weight (DW) of male Buzzards: M1, M2, M3 (*n* = 3) before and after stress event. Missing data points are due to missing or insufficient fecal samples.

**Figure 3:**
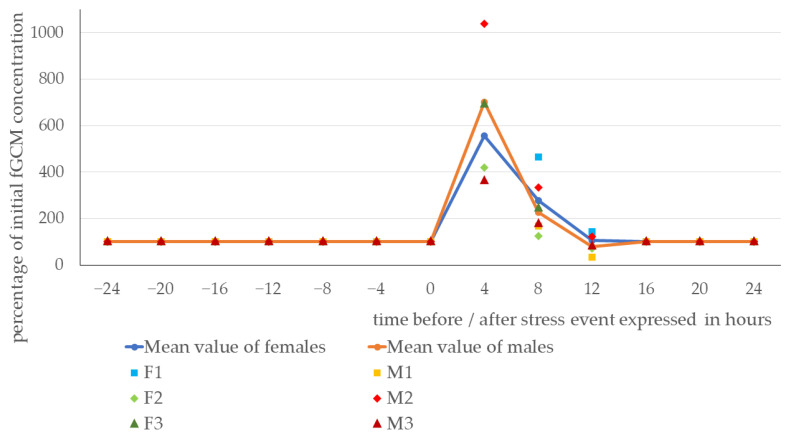
Variation in fecal glucocorticoid metabolite (fGCM) levels after the stress event in relation to the individual baseline (all values before the stress event are set to the average 100%). Male Buzzards: M1, M2, M3 and female Buzzards: F1, F2, F3. Missing data points are due to missing or insufficient fecal samples.

As demonstrated in Figure 1 and Figure 2, females and males showed a distinct increase in fGCMs during the first four hours after the stress event. From a baseline of 6866 ng/g dry weight (DW) in the females and 6920 ng/g DW in the males, the mean glucocorticoid metabolite concentration increased to 38,219 ng/g DW in females and 48,586 ng/g DW in males during the first few hours after the stress event (for details, see Table A1). The overall baseline using all animals was 6893 ng/g DW and the corresponding mean concentration during the first four hours after the stress event was 43,402 ng/g DW (increase to 629% in relation to the baseline). Expressed as the mean value of the percentage variation of the initial fGCM concentration, this indicated an increase to 557% in females and 702% in males (see Figure 3). As little as 8 h after the start of the stress event, the fGCM concentration dropped to an average of 279% (females) and 227% (males) and reverted to the basal level by, at the latest, 16 h after the start or 14 h after the end of the stress event.

### 3.3. Veterinary Examination during Stress Event

All six birds were very agile, as they showed combative behavior during the examination and demonstrated a good physiological condition and body condition score (BCS). Five of the six birds showed pressure marks on the feet. Four of them were classified as pododermatitis grade I and one (M2) showed signs of necrotic tissue alterations, grade IV pododermatitis (bumble foot). One bird (F3) had an old, healed fracture of the sternum and a shortened anterior wing skin (propatagium) on the left wing. Another (M3) had missing finger bones on the left hand and was therefore unable to fly. The blood test results were physiological in all Buzzards, except for the iatrogenic increase in aspartate aminotransferase (AST) and creatine kinase (CK) caused by the blood sample (for details, see Supplementary Materials).

### 3.4. Differences in Sexes and Time of Day

**Figure 4:**
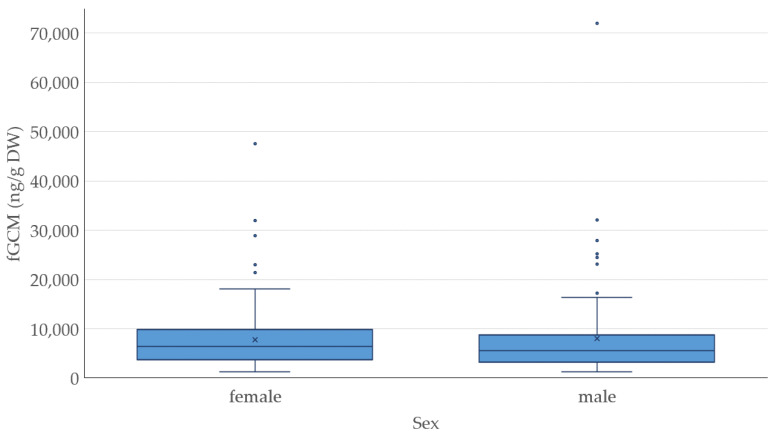
Comparison of fecal glucocorticoid metabolite (fGCM) levels in ng/g dry weight (DW) between the sexes.

No statistically significant differences could be found between the sexes (*p* = 0.903), as seen in Figure 4. For both sexes, the baseline values were around 7000 ng/g DW (6866 females, 6920 males) and both sexes also reacted with a similar increase (5.5 to 7-fold) during the stress event.

**Figure 5:**
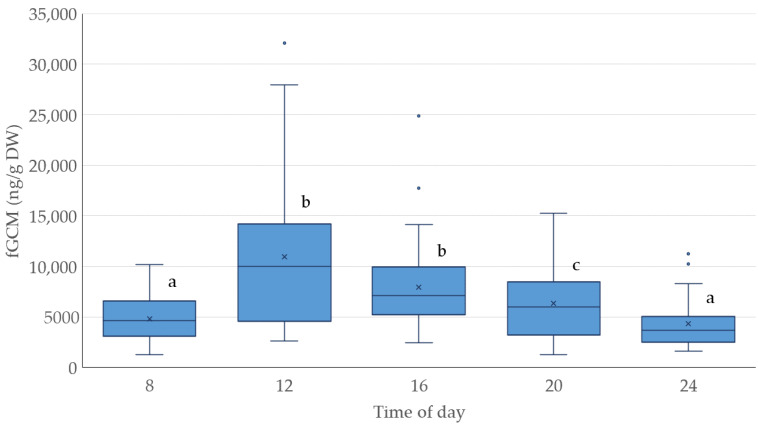
Diurnal variation in fecal glucocorticoid metabolite (fGCM) levels in ng/g dry weight (DW). Significant differences between times of day are indicated by different letters.

Significant differences between different times of the day could be found. Therefore, the means at the times were compared (see Figure 5 for details). When comparing the same times between different days, only the differences for 16 and 20 h compared with the day of the event (day 4) were statistically significant (range of *p*-value from *p* < 0.0001 to *p* < 0.05; exception: 20 h at day 1 did not differ significantly from the same time at day 4).

### 3.5. Stability of Fecal Glucocorticoid Metabolite Concentrations Post-Defecation

**Figure 6:**
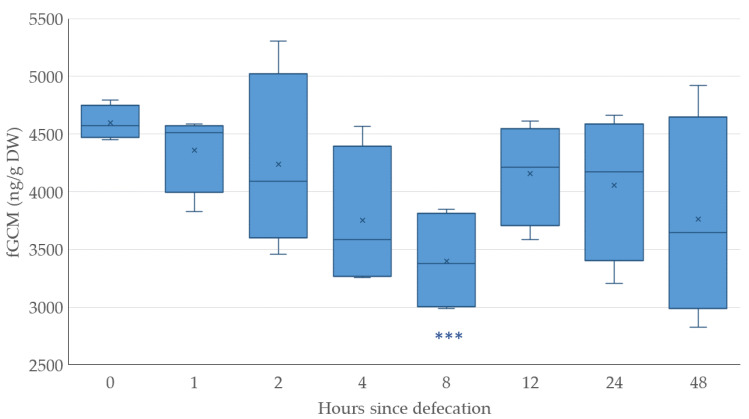
Post-defecation changes of immunoreactive fecal glucocorticoid metabolite (fGCM) levels in ng/g dry weight (DW) induced by degradation. Significant differences in comparison to the control 0 are indicated by asterisks (Dunnett’s test, *** = highly significant (*p* ≤ 0.001)).

The data showed a drop in fGCM concentration in the first 8 h after defecation by a total of 26%, in the following 4 h again approaching the original value (91%), thereafter once again dropping to 75% between hours 12 to 48. The difference between 8 h versus 0 h was highly significant (*p* ≤ 0.001), as seen in Figure 6.

## 4. Discussion

The aim of the study was to validate an enzyme immune assay (EIA) for the non-invasive measurement of glucocorticoid metabolites as a stress indicator in the Common Buzzard, and to establish data on possible sampling and processing intervals for further studies by testing the stability of the fGCM concentration post-defecation.

In our study, the Cortisone EIA was chosen to measure the physiological stress response and recovery following a stressful event for both males and females. In other studies involving birds, often, a commercial Corticosterone EIA [4,29,41,42,55,56,57,58] or radioimmunoassay (RIA) [35,38,48,59,60,61] was used. These were originally developed to measure plasma corticosterone and can therefore detect fGCMs only via cross-reactivity [49]. Although RIAs are very precise, they require radioactive substances, which are not available for metabolites [49]. Newly established assays can be carefully adapted to each species by developing group-specific antibodies, taking into account inter-individual and sex differences [62]. Radio-metabolism studies help to detect excretion patterns in different species [16]. Rettenbacher et al. successfully established a cortisone EIA for chickens [31]. Nonetheless, even in closely related species like the chicken and Japanese Quail, using the same assay is not always promising [32]. Therefore, it is important to test different assays for every species [26].

As reviewed in the work of Touma and Palme (2005), most fGCM studies are validated via physiological validation using an ACTH challenge test and sometimes a dexamethasone suppression test [26]. The ACTH challenge test provides a standardized and reliable method to measure an increase in fGCMs. Since this would have required the Buzzards to be captured and restrained for injections in the same way as the biological validation, the fGCM concentrations would not have been solely due to the application of ACTH. A purely biological validation gives us the opportunity to see to what extent handling stress affects Buzzards. Other studies with only biological validations [28,58,63,64,65] proved the reliability of this type of validation, e.g., Puerto Rican Parrots showed a significant elevation of fGCMs after capture and restraint [58]. Biological validations using a handling and capture stress event have also been successfully used in various studies for fGCM measurement [4,28,33,64,66,67,68].

Our tested baseline levels with around 7000 ng/g dry weight (DW) were high in comparison with other birds’ baselines, like those of the Greater Rhea [61], Goshawk [42], Northern Spotted Owl [35], Humboldt Penguin [33], or American Kestrel [40]. In those studies, either the wet weight, the dry weight, or the absolute weight (ng/g feces) was determined; therefore, a direct comparison between these units is not expedient. However, the dry weights are usually more suitable and more comparable with each other, as the consistency of feces can vary greatly. It should be noted that the sampling had to be performed inside the aviary, as it was not possible to collect the samples from the outside. This definitely influenced the behavior of the animals, despite their previous habituation, as they ran or flew back and forth when people entered the aviary, and this may also have influenced the fGCM levels. Although the Buzzards had lived in permanent husbandry for at least 11 months up to 8 years prior to the study, they were not used to humans entering the aviary multiple times a day. Other studies showed that wild animals can acclimatize to human contact or disturbance [69]. Therefore, a habituation period is recommended to allow them to get used to the new environment and sampling procedure. Furthermore, although the basal values of Buzzards habituated to human care are probably more comparable to the basal values of free-ranging Buzzards undisturbed by humans than those of freshly captured or injured Buzzards brought to the wildlife center, the values cannot simply be transferred. Studies collecting fecal samples from free-ranging undisturbed Buzzards are needed to verify the basal values for this species [51].

In contrast to many other studies, we did not find significant differences in the baseline glucocorticoid levels or stress responses between females and males. In rats [70], chickens [31], Puerto Rican Parrots [58], Northern Spotted Owls [35,37], Blue Wildebeest [71], and Sea Lions [72], males tend to have significantly higher baseline values or stress responses than females. However, in mice [73], hares [74], and African Wild Dogs [75], females tend to have higher baseline glucocorticoid concentrations compared to males. These differences between the sexes are possibly due to different metabolic and excretory patterns and are often also dependent on the breeding season [26,27,35,40,55,58,59,76,77,78,79,80,81]. Similar to the Buzzards, studies in Mourning Doves [48] and Red Deer [82] also showed no sex differences. Further studies with a larger sample size would be recommended to confirm this. To determine whether there is a seasonal change in fGCMs in male and female Buzzards, further research needs to be conducted at different times of the year, especially during the breeding season.

Differences in hormone concentrations over the course of the day are usually due to a pulsatile physiological release of steroid hormones and often show high levels at the start and/or end of the active time of day [26,28,53,70]. In our study, the birds had a peak in their fGCM concentration between 08:00 and 12:00, shown in the significant test results from 12:00 vs. 08:00, 20:00, and 24:00, respectively. However, in this study, the 12:00 sample contained all the feces from 08:00 to 12:00 and the 08:00 sample contained all the samples from midnight to 08:00. This would support the findings that the values are highest at the beginning of the active daytime. Nevertheless, it must be questioned whether the 08:00 samples were confounded by the significant drop in fGCM concentration after eight hours shown in the results of the stability experiment. Even though the weather during sampling was consistently cool and thus did not correspond to the tested room temperature of 18–22 °C, and the animals sleep at night and defecate very little during this time, metabolization of fGCMs by bacterial enzymes may have caused the 08:00 values to be very low. For future studies it is important that samples older than 4 h should be excluded from analysis, that sampling should take place at set times, and that sampling does not vary over the course of the day.

Following literature, diet seems to have an impact on herbivores depending on the fiber content, which influences the food intake and gut passage time [59,78,83], but carnivores like raptors have a short gut and no great diversity in the composition of their diet. However, it is questionable whether the fGCM of the prey can also be found in the feces of the predators [27].

Additional external stressors cannot be completely ruled out in the experimental setup. The aviaries were located in a remote environment, there was no visual contact with humans or other animals, except during feeding and sampling, and the acoustic perception of people, vehicles and bird calls was comparable to the “background noise” in the wild. However, the tested animals were able to hear the vocalization and movements of the other two subjects in the neighboring aviaries. The latter may have led to additional stress, as previous studies have indicated that fGCM levels increased due to the presence of competitors [28,84]. Environmental stressors such as food deprivation, high temperatures, or poor habitat conditions and human disturbance such as proximity to roads or exposure to tourists have negative effect on stress levels, as described for chickens [85], Willow Tits [86], Magellanic Penguins [69], and White-tailed Sea Eagles [43], although they seem to habituate to the presence of humans [36,87]. This underlines the importance of a habituation period and standardized environment when conducting experiments with captive wildlife and must be taken into account when taking samples from free-ranging species.

The stability of fGCM concentration post-defecation differs between species and should therefore always be taken into account [50,78]. This is necessary for planning the sampling interval in further studies with free-living Buzzards. Bacterial enzymes can cause the fGCM concentration in feces to continue to rise or fall after a certain time [14,88]. In White-tailed Sea Eagles, the stability of urine GCM concentration lasted for 9 days [43], in the Nile Crocodile the fGCM concentration in feces remained stable for 72 h at ambient temperature [89], while in Grey Mouse Lemurs it remained stable for at least 10 h [64], in Cheetahs for 22 h at 0–4 °C [90], in Blue Wildebeest for 8 h [71], and in Leopards even up to 6 days post-defecation [91]. In the present study, glucocorticoid concentrations remained relatively stable with a variation of up to 18% within the first 4 h. After that, the measured concentration dropped significantly in the 8 h subset. This must be considered in further studies on Common Buzzards when planning sampling intervals. For sampling in the field, this means that collected samples remain stable at room temperature for at least 4 h and do not need to be frozen immediately. In order to optimize the fecal sample collection in the field, a more detailed stability experiment with smaller time intervals between 4 and 8 h after defecation is needed to determine the exact time point at which the alterations in GCM concentrations occur.

Although we could not detect any significant inter- or intra-individual variation, these have both been described by many authors previously [28,42,92,93]. Therefore, individual patterns in hormone metabolism should be kept in mind, especially when age and reproductive status are unknown. The total sample size should be large enough to detect outliers or differences. In addition, the total amount of feces should always be collected and homogenized before taking a subsample from it [93]. Palme et al. (2000) also suggested that individual variation can be reduced by expressing the increase in the stress response as a percentage of the baseline, thereby allowing the animal to appear as its own control [94].

Some studies used the whole dropping of the bird for GCM reading, as feces and uric acid collect in the cloaca and are excreted together [30,95]. Other studies showed that the excretion of GCM via the urine is much faster than via the gut and a collection of both can distort the results [32]. Therefore, it is mostly recommended to only collect the fecal part of the dropping [59]. Raptor droppings are usually very watery, so collection of feces without contamination with uric acid is virtually impossible. When collecting samples, the uric acid content should be recorded (low-grade to high-grade) so that any inconsistent values can be reconstructed later.

Another influencing factor may be the small sample mass in raptors’ feces. Tempel et al. (2004) and Hayward et al. (2010) stated that small masses of fecal samples result in a higher fGCM concentration [36,60]. Nevertheless, Goymann (2005) questioned those results and calculated that the correlation found may be spurious [59]. A special RIA was developed for Budgerigars [34]. However, very small sample quantities (less than 0.02 g) should be excluded from the analyses or mixed into pooled samples [51]. The sampling interval of 4 h chosen in our study proved to be reliable to achieve a sufficient total sample mass.

For further studies on Buzzards and other raptors, a system to collect samples without having to enter the aviary would be of great advantage. The sampling interval needs to be adjusted to diurnal variations and to the stability of the samples post-defecation. Possible seasonal variations and resulting differences between the sexes need to be explored further.

## 5. Conclusions

We successfully validated the non-invasive measurement of fecal glucocorticoid metabolites as a stress indicator in the Common Buzzard via biological validation. The Cortisone assay was chosen to measure stress hormone metabolites in the feces of Common Buzzards. A practicable habituation and sampling period, as well as a sampling interval, were therefore successfully established. The standardized stress event was suitable to trigger a sufficient stress response. Baseline- and stress response concentrations for capture and handling are available for further studies in wild Common Buzzards for comparison. We did not find significant sex differences, but diurnal variations in the subjects.

In summary, the results of this study provide a basis for future studies on stress in birds of prey during treatment and rehabilitation in wildlife centers. As the measurement of fecal glucocorticoid metabolites can be a reliable tool to analyze the stress impact of human interactions and a wide range of environmental factors on wild animals, these measurements may provide new insights for animal welfare, especially during hospitalization, treatment, and rehabilitation, for example, measuring stress levels in the different stations that are passed through in wildlife centers to improve the keeping of wild birds of prey. With this in mind, the baseline values of this investigation can be used for subsequent studies on Common Buzzards, and the methodology established here can be validated in further species.

## Data Availability

All raw data is provided in the Appendix A and Supplementary Materials.

## Supplementary Materials

The following supporting information can be downloaded at: https://www.mdpi.com/article/10.3390/ani14081234/s1, Table S1: Results of blood examination (*n* = 6) in female (F1, F2, F3) and male (M1, M2, M3) animals, Table S2: Results of X-rays for female (F1, F2, F3) and male (M1, M2, M3) animals (*n* = 6).

## Appendix A

**Table A1:** Results of biological validation in female (F1, F2, F3) and male (M1, M2, M3) Buzzards (*n* = 6, n.t. = not tested).

Hours before/after Stress Event	Females: fGCM (ng/g Dry Weight)				Males: fGCM (ng/g Dry Weight)			
	F1	F2	F3	Mean	M1	M2	M3	Mean
−76	1975	4402	6828	4402	1680	6776	4360	4272
−72	5367	5709	11,118	7398	3847	17,263	12,455	11,188
−68	9954	13,181	17,753	13,629	5683	24,858	5824	12,122
−64	3242	5111	15,258	7870	n.t.	10,459	7072	8766
−60	1922	3298	10,259	5160	2299	n.t.	5116	3707
−52	1279	5026	7890	4732	1347	5407	3738	3497
−48	3433	7864	12,224	7840	4401	15,913	10,385	10,233
−44	4765	5212	7141	5706	4074	14,125	7705	8635
−40	12,357	4402	7287	8015	2529	5560	3194	3761
−36	1715	2913	8082	4237	1602	3717	11,245	5521
−28	3362	1688	7873	4307	2795	5616	6144	4851
−24	6595	3402	6263	5420	4191	12,450	24,434	13,691
−20	7355	3781	7181	6105	4218	n.t.	7181	5700
−16	5648	6384	8609	6880	2332	n.t.	5475	3904
−12	2504	n.t.	5038	3771	1636	8280	4972	4963
−4	2446	2008	10,376	4943	2927	6196	6389	5170
0	3737	5497	18,094	9109	2586	16,375	16,341	11,767
4	n.t.	28,886	47,553	38,219	n.t.	71,970	25,201	48,586
8	31,986	8549	16,919	19,152	11,654	23,096	12,391	15,714
12	9846	4949	n.t.	7398	2304	8522	5620	5482
20	3810	2273	6602	4228	1494	8505	6367	5455
24	8554	2638	13,826	8339	2923	9663	11,271	7952
28	7764	2454	7568	5928	2796	6674	5886	5119
32	2309	1292	11,125	4909	2460	7890	7836	6062
36	3361	4587	n.t.	3974	2495	3611	5289	3799
44	6612	3472	6958	5681	3881	6237	5804	5307
48	5095	11,185	21,416	12,565	3161	14,354	16,232	11,249
52	7602	6398	9626	7875	3907	11,477	6872	7419
56	4360	8701	11,493	8185	2228	8402	5361	5330
60	2758	6319	n.t.	4539	3263	4249	4753	4088
68	4052	4793	10,198	6347	4471	7352	3012	4945
72	6726	13,259	23,007	14,330	2880	27,982	32,063	20,975
76	10,986	5241	12,809	9679	3098	10,616	6602	6772
80	6749	6487	11,658	8298	2445	6404	4204	4351
84	3457	4038	n.t.	3748	2258	4261	5031	3850

**Table A2:** Results of stability experiment of four sets (A–D) with eight subsamples each (1–8) of sampled Buzzard feces (*n* = 42).

Lab Number	Temperature °C	Recollected and Frozen after Hour	fGCM (ng/g DW)
A-1	20	0	4453
A-2	20	1	3828
A-3	20.5	2	5307
A-4	20	4	3874
A-5	20	8	3708
A-6	20	12	4078
A-7	21	24	3990
A-8	21	48	3818
B-1	20	0	4796
B-2	20	1	4529
B-3	20.5	2	4166
B-4	20	4	3258
B-5	20	8	3848
B-6	20	12	4351
B-7	21	24	4665
B-8	21	48	4923
C-1	20	0	4612
C-2	20	1	4499
C-3	20.5	2	4020
C-4	20	4	3300
C-5	20	8	3045
C-6	20	12	4614
C-7	21	24	4357
C-8	21	48	3479
D-1	20	0	4535
D-2	20	1	4589
D-3	20.5	2	3461
D-4	20	4	4568
D-5	20	8	2988
D-6	20	12	3584
D-7	21	24	3208
D-8	21	48	2827
