# Supplementary material for: Validation of Fecal Glucocorticoid Metabolites as Non-Invasive Markers for Monitoring Stress in Common Buzzards (Buteo buteo)
Source: Animals (Basel). 2024 Apr 19;14(8):1234. doi: 10.3390/ani14081234 (PMC11047616; doi:10.3390/ani14081234)
Supplement: Supplementary file 1 [file animals-14-01234-s001.zip › animals-2934973-supplementary.pdf]

Article

Validation of Fecal Glucocorticoid Metabolites as Non-Invasive Markers for Monitoring Stress in Common Buzzards (*Buteo buteo*)

Lara-Luisa Grundei [1,*], Tanja E. Wolf [2,3], Florian Brandes [4], Karolin Schütte [4], Fritjof Freise [5], Ursula Siebert [6], Chadi Touma [2] and Michael Pees [1]

[1] Department of Small Mammal, Reptile and Avian Medicine and Surgery, University of Veterinary Medicine Hannover, Foundation, Bünteweg 9, 30559 Hannover, Germany
[2] Department of Behavioral Biology, School of Biology/Chemistry, Osnabrück University, Barbarastr. 11, 49076 Osnabrück, Germany
[3] Mammal Research Institute, Faculty of Natural and Agricultural Sciences, University of Pretoria, Pretoria, 0028 South Africa
[4] Wildlife Rescue and Conservation Center, Hohe Warte 1, 31553 Sachsenhagen, Germany
[5] Department of Biometry, Epidemiology and Information Processing, University of Veterinary Medicine Hannover, Foundation, Bünteweg 2, 30559 Hannover, Germany
[6] Institute of Terrestrial and Aquatic Wildlife Research, University of Veterinary Medicine Hannover, Foundation, Bischofsholer Damm 15, 30173 Hannover, Germany
* Correspondence: lara-luisa.grundei@tiho-hannover.de

Supplementary Materials

Table S1: Results of blood examination (n=6) in female (F1, F2, F3) and male (M1, M2, M3) Common Buzzards.

Comprehensive metabolic panel (CMP)	*Standard value*	*F1*	*F2*	*F3*	*M1*	*M2*	*M3*
a-Amylase	- U/l	277.00	403.00	682.00	354.00	900.00	900.00
Cholinesterase	840-1702 U/l	2706.00	3780.00	3897.00	2922.00	2447.00	2385.00
Bile acids	20-118 µmol/l	2.52	19.71	13.51	3.90	7.15	3.20
Aspartate aminotransferase	50-105 U/l	463.80	716.10	843.90	355.80	439.90	329.90
Creatine kinase	357-850 U/l	970.00	1022.00	5052.00	560.00	1338.00	1808.00
Lactate dehydrogenase	625-1210 U/l	398.80	560.00	930.70	357.00	632.30	316.20
Total Protein	25-40 g/l	36.00	37.20	43.50	30.40	28.10	36.70
Glutamate dehydrogenase	- U/l	3.50	1.60	22.20	0.20	4.70	0.40
Uric Acid	326-675 µmol/l	405.00	691.00	696.00	132.00	221.00	232.20
Cholesterol	3.9-10.5 mmol/l	7.10	7.80	11.90	6.60	11.90	8.00
Triglycerides	- mmol/l	1.23	1.59	1.14	0.74	0.60	0.74

Phosphorus	0.77-2.1 mmol/l	0.50	0.40	0.20	0.20	0.40	0.40
Calcium	2.1-2.56 mmol/l	2.40	2.30	2.40	2.00	2.20	2.30
Sodium	153-164 mmol/l	161.00	155.00	156.00	159.00	166.00	150.00
Potassium	0.9-1.7 mmol/l	1.90	2.70	3.20	2.20	3.00	3.60

Table S2: Results of X-rays for female (F1, F2, F3) and male (M1, M2, M3) Common Buzzards (n=6); note small circular radiopaque artefact on the X-ray plate.

Animal ID	***Laterolateral***	***Ventrodorsal***
F1	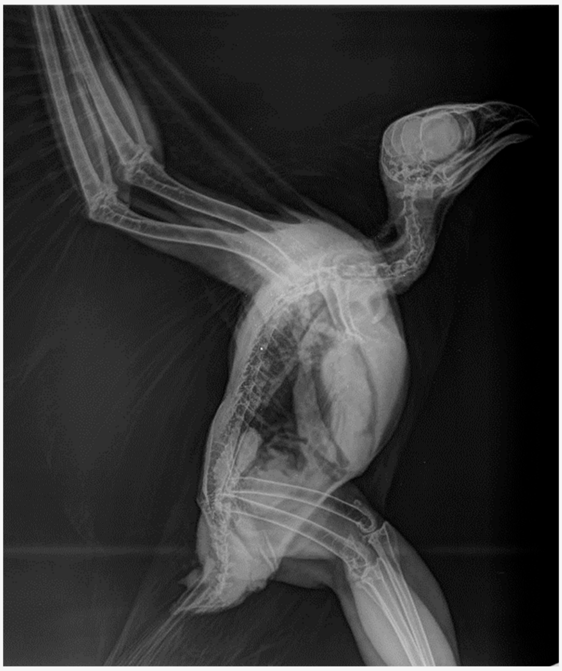	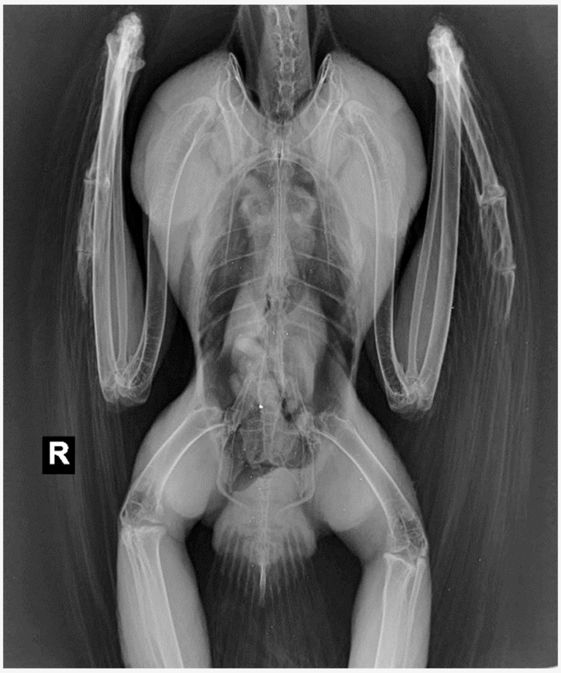

F2	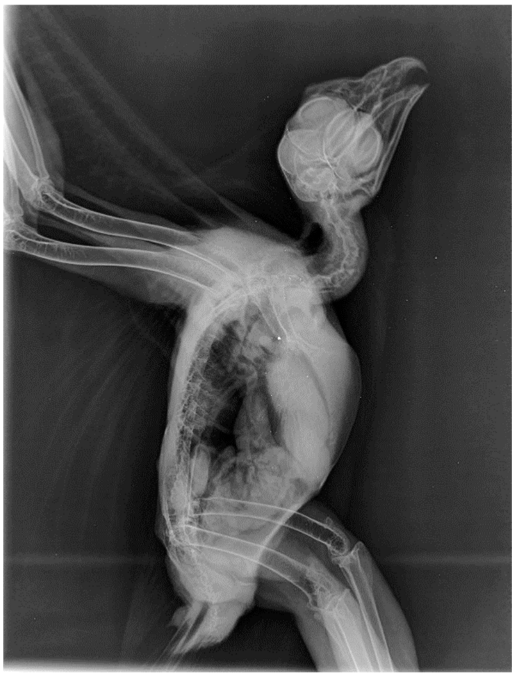	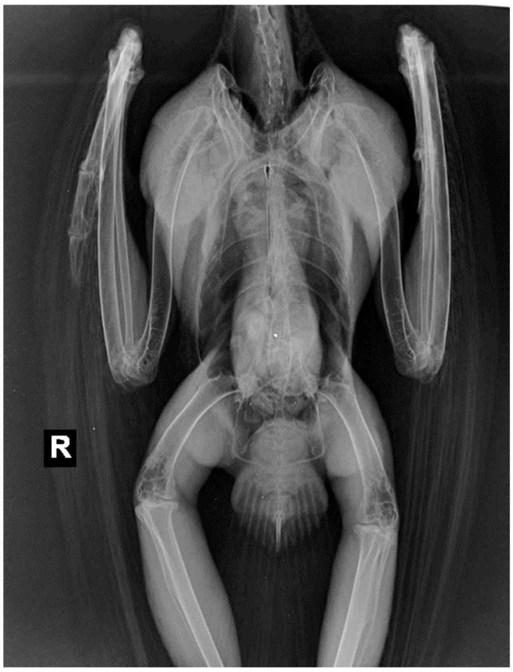
F3	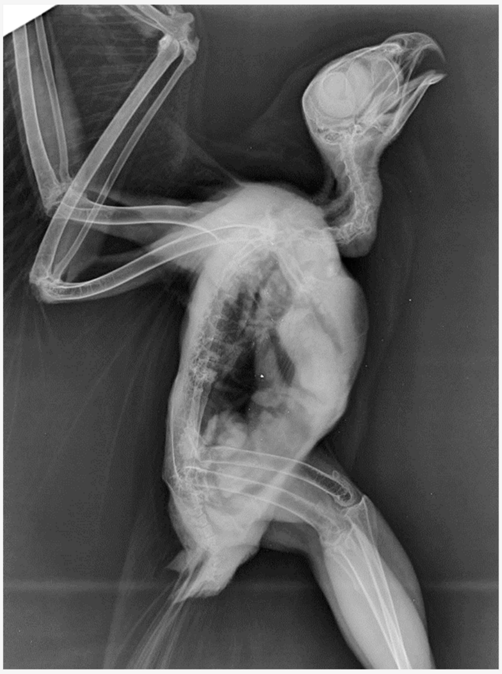	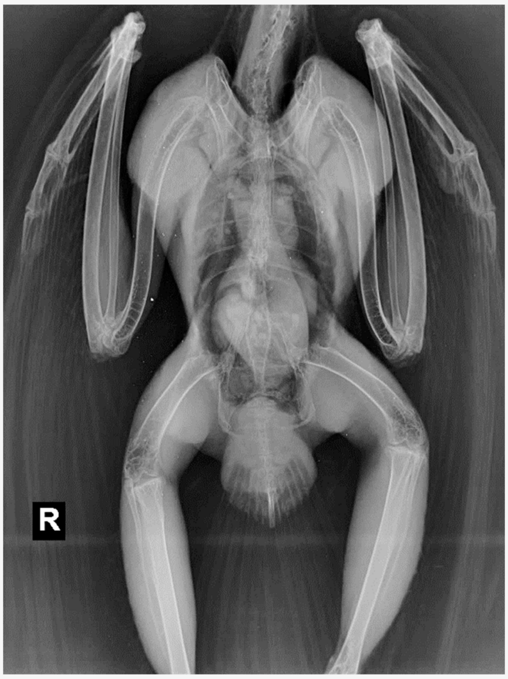

M1	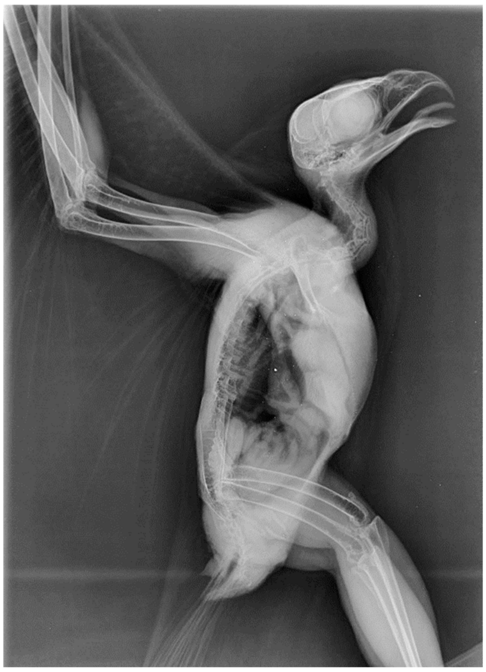	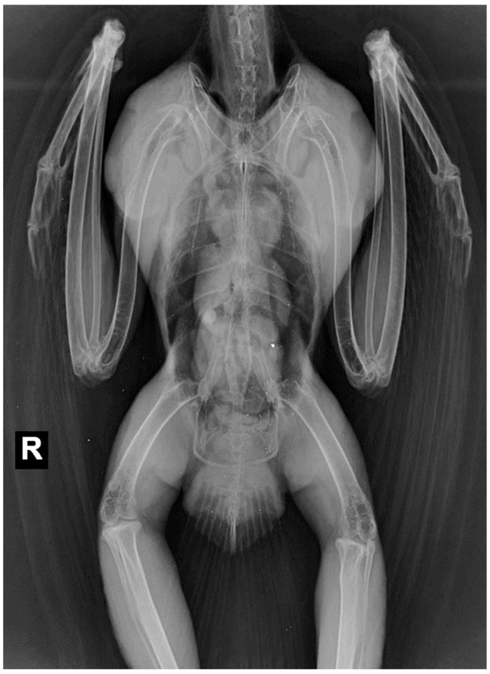
M2	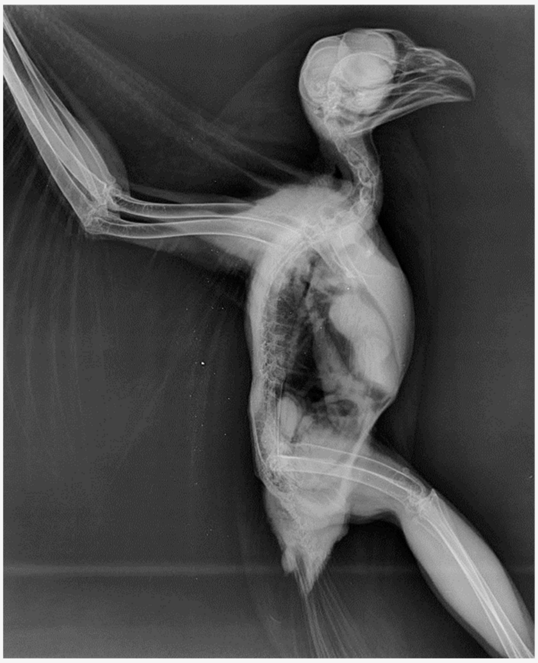	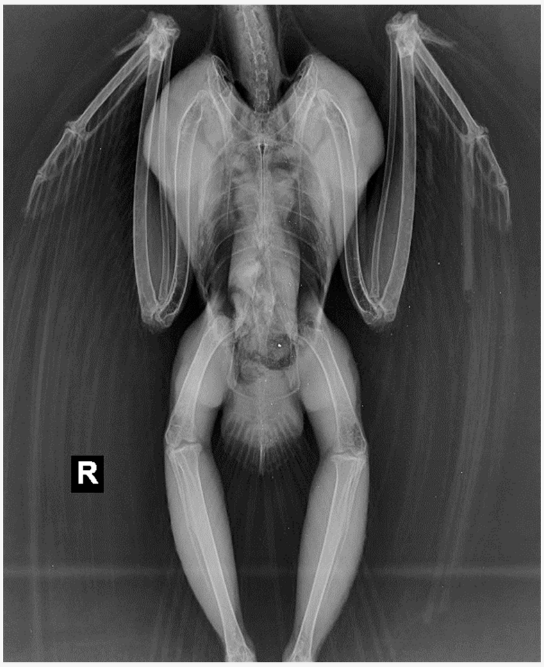

M3	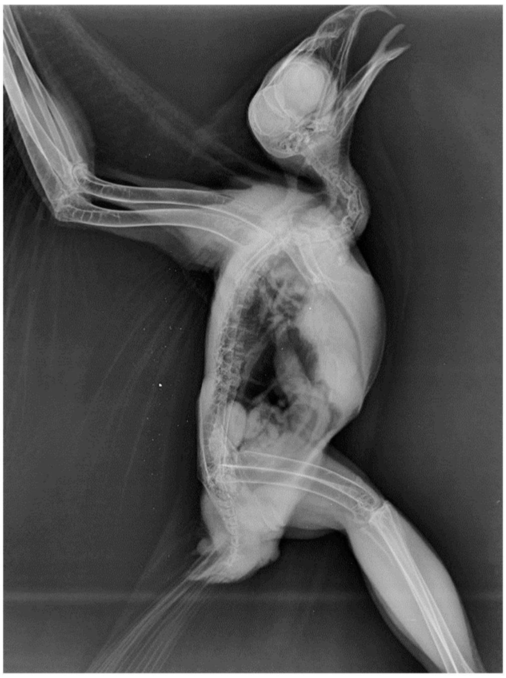	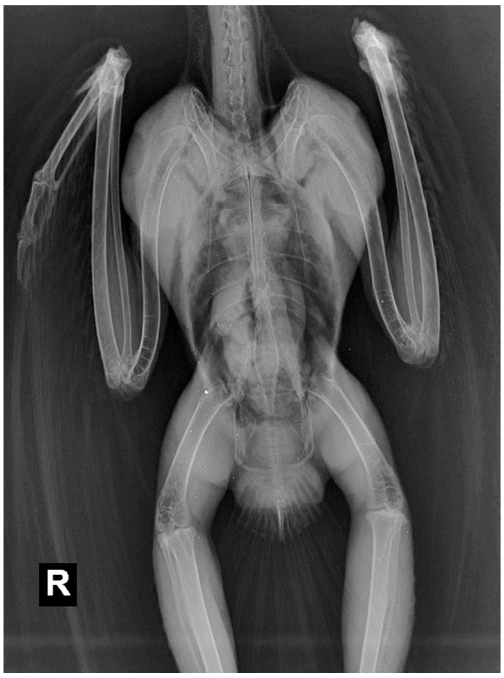
